# Ceramic Processing of Silicon Carbide Membranes with the Aid of Aluminum Nitrate Nonahydrate: Preparation, Characterization, and Performance

**DOI:** 10.3390/membranes11090714

**Published:** 2021-09-17

**Authors:** Esra Eray, Victor Manuel Candelario, Vittorio Boffa

**Affiliations:** 1 Department of Research and Development, LiqTech Ceramics A/S, Industriparken 22C, DK-2750 Ballerup, Denmark; vcl@liqtech.com; 2 Center for Membrane Technology, Department of Chemistry and Bioscience, Aalborg University, Fredrik Bajers Vej 7H, DK-9220 Aalborg Øst, Denmark; vb@bio.aau.dk

**Keywords:** silicon carbide membrane, ceramic processing, sintering additive, oily wastewater treatment

## Abstract

The development of a low-cost and environmentally-friendly procedure for the fabrication of silicon carbide (SiC) membranes while achieving good membrane performance is an important goal, but still a big challenge. To address this challenge, herein, a colloidal coating suspension of sub-micron SiC powders was prepared in aqueous media by employing aluminum nitrate nonahydrate as a sintering additive and was used for the deposition of a novel SiC membrane layer onto a SiC tubular support by dip-coating. The sintering temperature influence on the structural morphology was studied. Adding aluminum nitrate nonahydrate reduced the sintering temperature of the as-prepared membrane compared to conventional SiC membrane synthesis. Surface morphology, pore size distribution, crystalline structure, and chemical and mechanical stability of the membrane were characterized. The membrane showed excellent corrosion resistance in acidic and basic medium for 30 days with no significant changes in membrane properties. The pure water permeance of the membrane was measured as 2252 L h^−1^ m^−2^ bar^−1^. Lastly, the final membrane with 0.35 µm mean pore size showed high removal of oil droplets (99.7%) in emulsified oil-in-water with outstanding permeability. Hence, the new SiC membrane is promising for several industrial applications in the field of wastewater treatment.

## 1. Introduction

Membrane-based technologies have become a backbone of water and wastewater treatment applications because of their high separation efficiency, lower energy consumption, and small environmental impact [1,2]. At present, polymeric membranes are the most applied membrane material in water and wastewater treatment processes. However, low thermal and chemical stability, high fouling propensity, and limited working lifespan of polymeric membranes limit their application field. On the contrary, good chemical, thermal, and mechanical stability, long working lifespan, and low fouling tendencies of ceramic membranes make them ideal for several water and wastewater treatment applications, which cannot be operated efficiently by the polymeric membranes [3,4,5]. In this context, silicon carbide (SiC) membranes have gained special attention as an attractive porous material among the other ceramic membranes such as alumina [6], titania [7], and zirconia [8]. The main advantages of SiC membranes are directly related with to their outstanding chemical, thermal, and mechanical properties under harsh conditions and high flux. Thanks to these features, a significant research effort has been devoted to the development of SiC membranes for the treatment of various water and wastewaters such as oily wastewater, produced water, surface water, and swimming pool water [9,10]. 

SiC membranes are generally composed of a layer deposited on a porous support, which provides high flux and strong mechanical strength for the membrane layer. The choice of the right support material with well-defined characteristics helps to prepare good quality and reproducible SiC membranes. Porous supports used for fabrication of SiC membranes generally consist of alumina [11,12] or SiC [13,14]. Indeed, SiC is one of the most promising materials also as a membrane support, thanks to aforementioned superior properties. It should also be noted that the crack formation during the membrane fabrication process is minimized when the support and the membrane layers consist of the same material, because of the same thermal expansion coefficient [15].

In addition, the choice of the preparation method for the SiC membrane layer is also essential in order to benefit from all the advantages of SiC. The widely used methods for preparing SiC membrane layers are deposition of colloidal suspensions or deposition of pre-ceramic polymeric precursors. Polymeric precursor-based SiC membranes are mainly used for gas separation applications, as pyrolysis of these precursors results in dense layers [16,17,18]. When these membranes were applied for water treatment purposes, they showed low water permeability, which is around 0.06 L m^−2^ h^−1^ bar^−1^, due to the dense structure [19]. On the other hand, SiC membranes developed by deposition of colloidal suspensions method result in porous membrane structure, which generally is more suitable for water and wastewater treatment applications [20,21,22]. Deposition of colloidal suspension is also called ceramic processing. In this method, SiC slurry prepared from SiC particles dispersed in alcoholic or aqueous media with a polymer binder can be deposited on a porous support by dip-coating, spin-coating, and slip-casting. This is followed by the partial sintering of the green body. Even though this method is simple and conventional for fabricating SiC membranes, there are still challenges to address. A major obstacle is the high sintering temperature (up to 2100 °C) required due to the covalent nature of Si-C bonds. Another challenge is the use of a large number of organic additives and a large amount of organic solvents, which makes the fabrication process environmentally unfriendly.

The high sintering temperature has direct effect on the SiC membrane production cost. In order to reduce the membrane processing temperature and consequently develop economically competitive SiC membranes, the use of sintering additives in the formulation of SiC suspensions have been considered an effective approach. The majority of the low melting point sintering additives promote densification process through liquid phase by modifying grain boundaries and surface energies. In the literature, SiC ceramics were prepared using various metal oxides sintering additives such as Al_2_O_3_ [15,23], Y_2_O_3_ [24,25,26], ZrO_2_ [27,28], CaO [27,29], and MgO [30] at temperatures ranging from 2000 to 1300 °C. Moreover, some studies showed that the sintering temperature of the SiC ceramics can be further decreased. For example, Yang et.al [31] reduced the sintering temperature of SiC ceramic support to 1150 °C using sodium dodecyl benzene sulfate as a sintering additive. Similarly, Jiang et al. [32] prepared SiC membrane at a low temperature of 1000 °C using zeolite residue as sintering additive.

It is often reported that the incorporation of sintering additives into SiC suspensions impairs the chemical and mechanical properties of the resulting membranes as these additives form second phases located at the grain boundaries [33,34]. On the contrary, it was also found that the SiC membrane filtration performance could be effectively improved with the use of sintering additives. For example, Han et al. [29] have employed CaO as sintering additive and found that the bending strength of SiC membranes improved by 73% to 22.5 MPa. Moreover, the prepared membranes showed good resistance in acid and basic solutions at 80 °C for 96 h. Li et al. [35] added B_4_C in the formulation of SiC suspension in order to prepare SiC membrane by co-sintering at 1900 °C. They fabricated a SiC membrane layer with a pore size of 0.28 µm that tightly bonded to the SiC support and membrane showed attractive chemical and permeation properties.

In order to address the two main challenges (environmentally unfriendly fabrication process and high sintering temperature) in the ceramic processing of SiC membranes, herein, a new silicon carbide membrane with the use of aluminum nitrate nonahydrate as a sintering aid was fabricated on a porous SiC tubular support via ceramic processing. An environmentally friendly colloidal suspension was prepared in aqueous media using only one organic additive, which acted as binder. The surface morphology, pore size distribution, elemental distribution, and crystalline structure of the developed membrane were characterized. Moreover, the corrosion resistance and mechanical strength of the SiC membrane were studied to validate the robustness of membrane in acid and alkali environments. The performance of the membrane was then evaluated by measuring its pure water flux. Finally, the effectiveness of the as-prepared SiC membrane was evaluated by filtration of a simulated olive oil washing wastewater sample. 

## 2. Materials and Methods

### 2.1. Raw Materials

Commercially available sub-micron α-SiC powders with average particle sizes of 0.4 µm and 0.6 µm were obtained from ESK, Germany. Aluminum nitrate nonahydrate (ACS Reagent grade, ≥98%) was purchased from Sigma-Aldrich, Søborg, Denmark and was employed as a sintering additive. As binder, Optapix CS-76, polysaccharide dicarbonic acid polymer, was purchased from Zschimmer & Schwarz, Lahnstein, Germany. All chemicals were used as received. Multi-channeled SiC tubular supports with an average pore size of 15 µm and a porosity of 40% were supplied by LiqTech Ceramics A/S and used for SiC membrane preparation. The supports measured 305 mm in length, 25 mm in diameter, and contained 30 channels of 3 mm diameter. 

### 2.2. SiC Membrane Fabrication Process 

**Figure 1:**
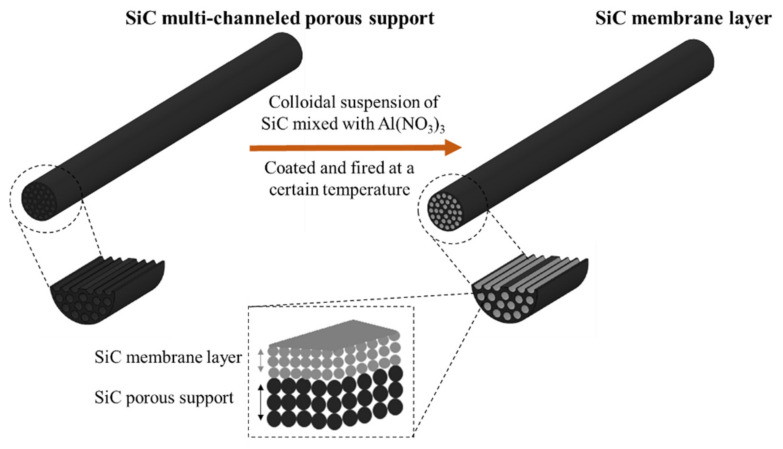
Schematic diagram of the fabrication process for SiC membranes.

For the preparation of SiC membrane layer on top of the SiC macroporous support, coating suspension was prepared using two α-SiC powders in different particle sizes, binder, sintering additive, and deionized water kept under continuous mechanical stirring with propeller (IKA RW 16). The suspension was prepared using the following sequential addition protocol. First, aluminum nitrate nonahydrate in concentration of 10^−4^ M was dissolved in deionized water. Then, 107.25 g α-SiC powder with 0.4 µm particle size was added to the mixture and mixed for 5 min. After that, 107.25 g α-SiC powder with 0.6 µm particle was slowly added and mixed for another 5 min. The pH of the suspension was adjusted to 10 by dropwise addition of 1M aqueous NH_3_. The suspension was then poured in polyethylene bottle and milled for 48 h with spherical 9 mm alumina milling beads to ensure homogenization. After milling, suspension was transferred into beaker and 1 wt% Optapix CS-76 was added, which is then stirred continuously at room temperature. The suspension was coated on macroporous SiC tubular support by dip-coating, followed by overnight drying at 40 °C. Subsequently, the membranes were sintered in the range of 1600 °C < T < 1900 °C for 4 h under argon atmosphere. As a last step, the sintered membranes were heat treated in an air furnace to remove the free carbon eventually formed in the pores. A schematic diagram of the fabrication of SiC membranes in this study is represented in Figure 1.

### 2.3. Characterization Methods 

The particle size distribution (PSD) of the coating suspension was determined by a Microtrac S3500 (Microtract Retsch GmbH, Haan, Germany) using dynamic light scattering (DLS) technique. The SiC powders were dispersed in the mixture of deionized water and aluminum nitrate nonahydrate with the ratio of 1:1 before test. 

Zeta potentials (ζ) of dilute SiC suspensions were measured using a Malver Zetasizer Nano ZS (Malvern Panalytical Ltd., Malvern, UK) to envisage the influence of the pH and suspension stability. The tracer solution was prepared by dispersing aluminum nitrate nonahydrate and the two α-SiC powders in 50 mL of distilled water. To adjust the pH of the tracer solution to 3–10, 0.1 M HCI or KOH solutions were used. Tracer solution was ultrasonicated for 5 min prior to zeta potential tests. Zeta potentials were measured 3 times to calculate the average value.

Scanning electron microscope, FlexSEM 1000 (Hitachi GmbH, Solna, Sweden), operating at acceleration voltage of 15 kV was used to characterize the surface and cross-sectional morphologies of the obtained membranes. The semi-quantitative elemental analysis of the samples was performed on the same SEM equipped with energy dispersive X-ray spectrometer (EDX). 

Capillary flow porometer (3P Instruments, Odelzhausen, Germany) was used to measure the mean pore sizes and pore size distributions of the SiC membranes. Prior to measurements, the pores of the membranes were filled with Porofil^TM^ (fluorinated hydrocarbon) wetting liquid having a surface tension of 16 dyn/cm. The wet curve was obtained by measuring air flow rate through the sample with increasing pressure. Then, the dry curve was measured by increasing the air pressure through a dry membrane sample. 

The crystalline structure of the SiC membranes was analyzed by X-ray diffraction using an Empyrean diffractometer (PANAnalytical, Eindhoven, The Netherlands), operated at 45 kV and 40 mA with Cu-Kα radiation (λ = 1.5418 Å). Scans were carried out in the 2θ range of 20–90°, using steps of 0.05°.

For the chemical resistance tests, the SiC membranes were separately dipped into a 0.25 mol/L H_2_SO_4_ and 0.25 mol/L NaOH at 60 °C. The samples were soaked in the respective solutions for 30 days and then thoroughly rinsed with deionized water for 1 h and dried in an oven at 40 °C for 24 h. Each week the pore size distribution of the membranes was measured, and their surface morphologies were evaluated to see if there is any change. 

Scratch tests were performed using a Taber 5900 reciprocating abraser (Taber Industries, North Tonawanda, NY, USA) equipped with a conical diamond tip with angle of 120°. The loads applied for each membrane at a constant speed of 1 cycle per minute was 1, 3, 5, and 10 N and the scratch widths were measured by SEM. Three different locations of each sample were measured for each load to obtain the average. 

The surface wettability of membranes was analyzed at ambient temperature using a contact angle goniometer (Attention theta, Biolin Scientific, Gothenburg, Sweden). Contact angles of the membranes were recorded by a OnePlus 6 camera (Sony IMX 519 image sensor, Tokyo, Japan), where 4 µL water droplets were dropped on the surfaces. At least two measurements were done on duplicated samples and the obtained contact angle results were averaged.

### 2.4. Membrane Filtration Performance 

The SiC membranes were tested for pure water permeance in a commercial cross-flow filtration setup (schematics in Ref. [20]) obtained from LiqTech Ceramics A/S, Denmark. The pure water flux of the SiC membranes was studied as a function of the transmembrane pressure ranging from 0.1 to 0.7 bar under cross flow of 1527 L/h for 15 min. Membrane flux (J) and permeability (Q) were determined using Equations (1) and (2) [36]:

(1) $$J=\frac{V}{\mathrm{At}}\;$$

(2) $$\;Q=\frac{V}{\mathrm{AtP}}\;$$

where V is the volume of the permeate (L), A is the effective membrane filtration area (m^2^), t is the filtration time (h), P is the transmembrane pressure (bar).

The filtration of model olive oil washing wastewater samples was also performed on the aforementioned cross-flow filtration setup. In order to prepare a stock emulsion of olive oil in water, 6 mL olive oil and 1.2 g sodium dodecyl sulphate (SDS) (Sigma-Aldrich, St. Louis, MO, USA) were dispersed into a 4 L deionized water with propeller (IKA RW 16) at 1200 rpm for 1 h. Then, the mixture was ultrasonicated (Hielscher Ultrasonic Homogenizer UIP2000hdT) for 1 h to form a milky white emulsion. Afterwards, the emulsion was diluted to 20 L with a concentration of 400 ppm for the filtration experiments. The feed was fed continuously into the system at a constant flux of 150 L m^−2^ h^−1^ and 0.08 bar constant transmembrane pressure for 1 h. Recovery factor set to 90%. After filtration, the permeate was collected and the retentate was not recycled back to the feed tank. Membrane performance was evaluated by the permeability and the removal of the oil and grease. The oil and grease content of the feed and permeate water were analyzed by a FTIR spectrophotometer (IRAffinity-1S, Shimadzu, Columbia, MD, USA). The membrane retention rate of oil and grease was calculated by Equation (3) [36]:

(3) $$R=\frac{C_{f}-C_{p}}{C_{f}}\times 100\%\;$$

where $C_{f}$ and $C_{p}$ are the oil concentrations (mg/L) in the feed and permeate, respectively.

## 3. Results and Discussion 

### 3.1. Characterization of Coating Suspension 

**Figure 2:**
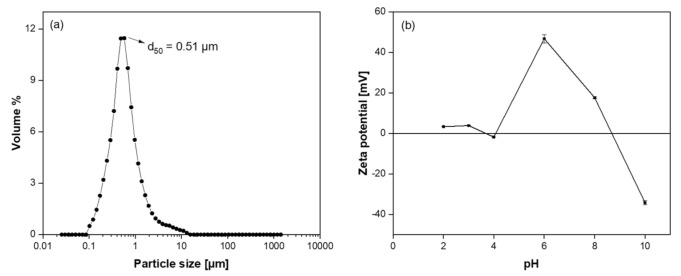
(**a**) Particle size distribution of the coating suspension and (**b**) Zeta potential as a function of pH at the surface of the diluted suspension.

The particle size and particle size distribution of the coating suspension play a critical role in preparing a defect-free membrane layer. For example, if the particle size distribution of the coating suspension is too broad and/or the particles are too large, the chance of defect formation at the membrane surface would be unavoidable during thermal treatment process. On the other hand, the particle size of the coating suspension must be large enough in order not to penetrate into pores of the support [19,37]. The particle size distribution of the SiC coating suspension is presented in Figure 2a. It shows that the coating suspension has a narrow pore size distribution with an average size of 0.51 µm, which indicates the colloidal suspension is suitable for coating on porous SiC support.

Another critical parameter for preparing defect-free membrane is the colloidal stability of the coating suspension, which here was evaluated using ζ- potential analysis. Figure 2b shows the ζ-potential of diluted suspension of SiC powders with aluminum nitrate nonahydrate, measured in the pH range of 2–10. The ζ-potential of the suspension at lower pH showed a similar behavior as unmodified SiC particles, followed by a maximum value at pH 6 were the charging effect from the absorbed hydrolyzed Al^3+^ species, which is more significant [38]. After reaching this maximum value, ζ-potential decreased from +46.7 mV to −34.2 mV and the isoelectric point of the suspension, i.e., the pH at which the net charge on the particle surface is zero, was determined to be approximately pH 9, with a similar curve tendency as Al_2_O_3_ particles. The surface of the particles was negatively charged above pH 9 and at pH 10 the ζ-potential of the SiC particles was −34.2 mV. Particles having large ζ-potential, greater than ±30 mV, are generally considered stably suspended [39]. Although the suspension was stable at pH 6 and 10, pH value of 10 was selected as the working pH for preparing the membrane in order to avoid precipitation of Al(OH)_3_ that occurs at pH 6 [38].

### 3.2. Structural Characterization of SiC Membrane

**Figure 3:**
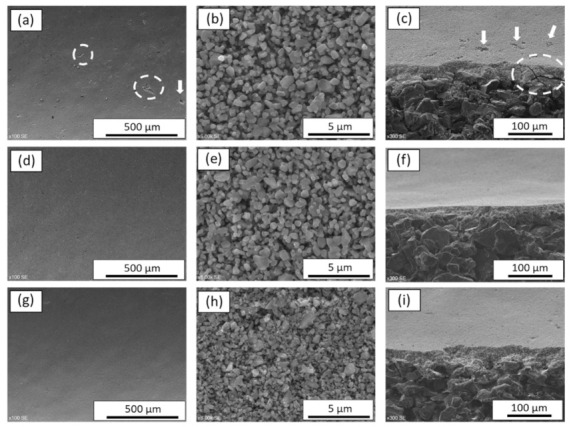
SEM images of surface and cross-section of the SiC membranes as a function of sintering temperature: (**a**–**c**) membranes at T_max_; (**d**–**f**) membranes at T_max_-200 °C; and (**g**–**i**) membranes at T_max_-300 °C.

The prepared SiC colloidal suspension with the aid of aluminum nitrate nonahydrate was coated on multi-channeled SiC tubular supports by dip-coating technique and sintered at the three different temperatures, in the range of 1600 °C < T < 1900 °C, for 4 h under argon atmosphere. The surface and cross-sectional morphologies of the developed SiC membrane with respect to sintering temperature are summarized in Figure 3. For confidentiality reasons, hereafter, the highest sintering temperature will be referred as T_max_, whereas the other two temperatures will be referred as T_max_-200 °C and T_max_-300 °C, respectively. 

When the membrane was sintered at the highest temperature of T_max_, macro-defects such as pinhole and crack were observed on the membrane surface and its cross section as can be seen in Figure 3a,c. At this temperature, the connection between the grains was clearly visible as represented in Figure 3b. The formation of macro-defects occurred probably because of the high sintering temperature that causes higher densification of the membrane layer with respect to the green body and, consequently, crack formation [20]. When temperature was decreased to T_max_-200 °C and/or T_max_-300 °C, no significant differences in membrane surface (Figure 3d,g, respectively) nor cross section (Figure 3f,i, respectively) were observed in terms of defect formation. In both cases, crack-free and homogeneous membrane layers with a thickness of 27.3 ± 0.9 µm for the membranes sintered at T_max_-200 °C and 29.4 ± 0.1 µm for the membranes sintered at T_max_-300 °C were obtained, and there was no visible infiltration. However, at T_max_-200 °C a good connection was formed between the SiC particles and thus an interconnected pore structure was formed, as presented in Figure 3e. On the contrary, when the sintering temperature was further decreased to T_max_-300 °C there was no apparent connection between SiC particles and less microstructural coarsening was observed on the membrane surface compared to membranes sintered at T_max_ and T_max_-200 °C, as can be seen in Figure 3h. The purpose of incorporating aluminum nitrate nonahydrate as a sintering additive was to reduce the high sintering temperature that is required by the conventional SiC membrane fabrication process. During sintering, aluminum nitrate nonahydrate melts, forming a liquid phase that promotes mass transfer at an atomic level [40,41]. Therefore, aluminum nitrate nonahydrate helps the sintering of SiC particles at lower temperatures in respect to the conventional SiC membrane synthesis. Except for the defect formation observed for the membrane sintered at T_max_, the surface morphologies of the membranes in terms of sintering behaviors of the SiC particles at T_max_ (Figure 3b) and T_max_-200 °C (Figure 3e) were similar. Hence, it was possible to decrease the sintering temperature down to T_max_-200 °C and to obtain defect-free membrane with the aid of aluminum nitrate nonahydrate. T_max_-200 °C was selected as an optimum sintering temperature for fabricating SiC membranes. 

**Figure 4:**
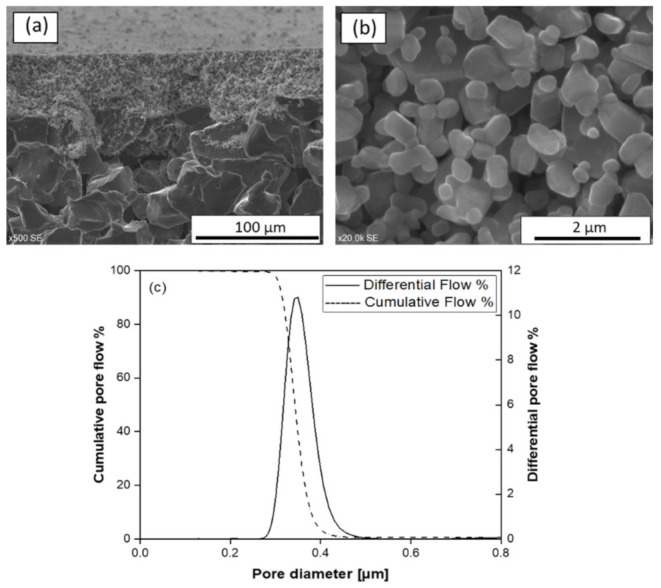
(**a**,**b**) SEM images of surface and cross-section of final SiC membrane sintered at T_max_-200 °C, respectively and (**c**) Pore size distribution of final SiC membrane.

In order to remove the residual carbon that may remain in the pores, a surface oxidation step was carried out at a temperature below 800 °C in air atmosphere. SEM micrographs of the cross-section and surface of the final membrane after the oxidation step are shown in Figure 4a,b, respectively. No significant difference on the surface morphology was found between sintered and oxidized samples, although surface roughness seems to be slightly reduced after the oxidation step.

Figure 4c shows the pore size distribution curve of the final SiC membrane. The membrane has narrow pore size distribution ranging from 0.3 µm to 0.5 µm with a maximum at 0.35 µm. Such sharp pore size distribution can be attributed to the uniform particle size of SiC particles and their uniform distribution in colloidal suspension [42]. In order to verify reproducibility, four independent samples were analyzed. All the samples showed no pores larger than 0.5 µm and the mean pore size value had a standard deviation ±0.6%.

**Figure 5:**
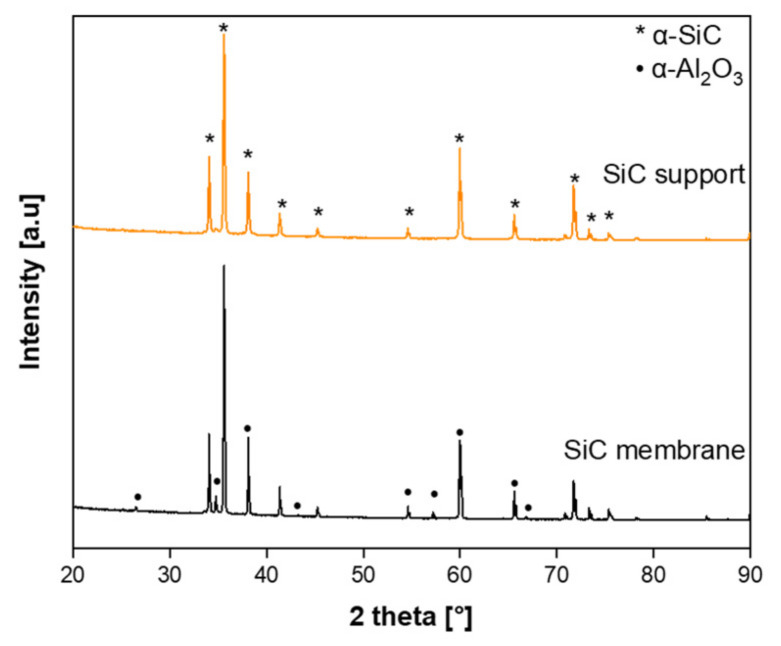
XRD patterns of the bare SiC support and the prepared SiC membrane layer on a SiC support.

The crystalline structure of the final membrane was analyzed by the XRD investigation. The XRD patterns of the bare SiC support and the prepared SiC membrane are shown in Figure 5. The observed diffraction peaks for both SiC support and membrane demonstrated characteristic of the hexagonal α-SiC crystalline structure (6H, α-SiC) [43]. The diffractions peaks found at 2θ = 25, 35, 37.5, 43, 52, 57, 61.5, 66, and 68° in the SiC membranes could be attributed to the (012), (104), (110), (113), (024), (116), 018), (214), and (300) reflection of the α-Al_2_O_3_ crystalline structure, respectively [44,45]. This is due to the reaction of aluminum nitrate nonahydrate to form α-Al_2_O_3_ at temperatures above 1100 °C. The peak intensity of α-Al_2_O_3_ was slightly weaker, indicating its poor crystallinity induced by aluminum nitrate nonahydrate.

**Figure 6:**
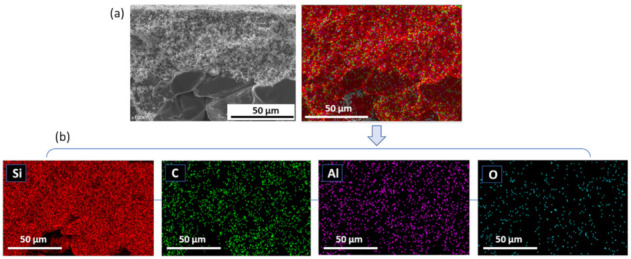
(**a**) EDX mapping of the final SiC membrane cross-section and (**b**) Single element mapping of the final SiC membrane cross-section.

To confirm the presence of aluminum oxide and to evaluate the potential of particle penetration within the SiC support pores, EDX mapping of cross-section of the final SiC membrane was performed. As shown in Figure 6a,b, Al-containing domains are uniformly distributed within the membrane layer without agglomeration. Al-containing domains are also detected in some part of the porous SiC support. This observation indicates that some of the sintering additive infiltrated into the macroporous structure of SiC support to a lesser extent. 

### 3.3. Chemical and Mechanical Stability 

Following the successful fabrication of SiC membrane with the aid of aluminum nitrate nonahydrate and characterization in terms of surface morphology, pore size distribution, elemental distribution, and crystalline structure, we also studied the chemical and mechanical behavior of the SiC membranes to check their robustness. The chemical stability of the membranes was analyzed by measuring pore size distributions, surface morphologies, and elemental compositions before and after exposure to 30 days corrosion resistance test. In particular, SiC membranes were separately dipped into 0.25 mol/L H_2_SO_4_ and 0.25 mol/L NaOH at 60 °C. The mechanical stability of the membranes before and after the stability test was examined by means of scratch test.

**Figure 7:**
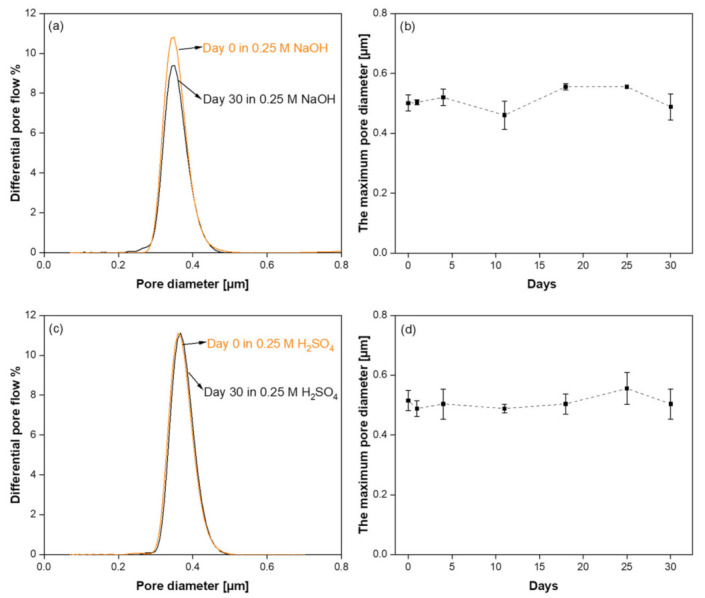
(**a**) Pore size distributions of final SiC membrane before and after alkali corrosion; (**b**) The change of the maximum pore size of SiC membrane exposed to alkali corrosion; (**c**) Pore size distributions of final SiC membrane before and after acid corrosion; and (**d**) The change of the maximum pore size of SiC membrane exposed to acid corrosion.

Figure 7a,c compare the pore size distribution curves of the SiC membranes before and after the corrosion resistance test in alkali and acid solutions, respectively. The narrow pore size distribution of the samples nearly remained at their initial range with pores between 0.2 and 0.5 µm, when exposed to acid and alkali after 30 days. Pore size measurements were repeated on two samples to verify reproducibility. The maxima of the pore size distribution curves were found 0.34 ± 0.02 and 0.35 ± 0.02 µm before and after alkali corrosion, respectively. In the case of acid corrosion, the pore size of the sample was 0.36 ± 0.03 µm before exposure and 0.37 ± 0.04 µm after exposure. During 30-day corrosion tests, the pore size distribution of the membranes was measured periodically in order to see if there is any change in the pore size. Figure 7b,d show the maximum pore size of the SiC membranes as a function of exposure days in alkali and acid solutions, respectively. It was observed that the maximum pore size of the membranes separately immersed into acidic and alkali solutions had not significantly changed, indicating the pore structures of the membrane were stable after 30 days with no evidence of defect formation in the membrane top-layer. 

**Figure 8:**
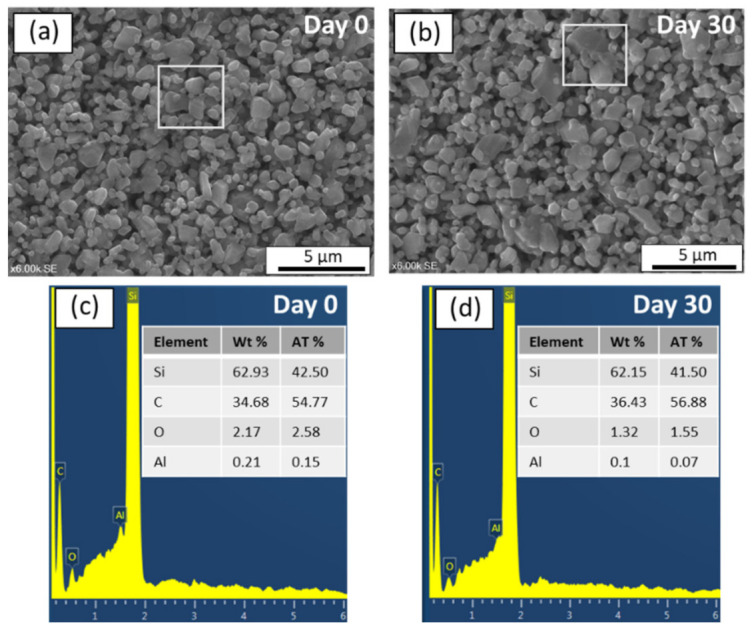
(**a**,**b**) SEM images of SiC membrane before and after alkali corrosion, respectively and (**c**,**d**) Chemical composition of SiC membrane layer before and after alkali corrosion, respectively.

In order to investigate the morphological changes before and after corrosion, SEM and EDX analysis were also conducted. As can be seen in Figure 8a,b, SEM image of the surface of an alkali exposed sample after 30 days remained identical to that of unexposed sample. Moreover, the EDX spectrums with chemical compositions of the membrane layers before and after alkali resistance test were given in Figure 8c,d, respectively. The EDX results of region highlighted in Figure 8a indicate the following elemental composition on the membrane surface before subjected to alkali: Si (62.93 wt%), C (34.68 wt%), O (2.17 wt%), and Al (0.21 wt%). After immersion in alkali for 30 days, the elemental composition of the membrane region highlighted in Figure 8b was: Si (62.15 wt%), C (36.43 wt%), O (1.32 wt%), and Al (0.1 wt%). Since there were no significant changes in the elemental composition on the membrane surface, these results demonstrate that SiC membrane fabricated in this study is stable in 0.25 mol/L NaOH for 30 days.

**Figure 9:**
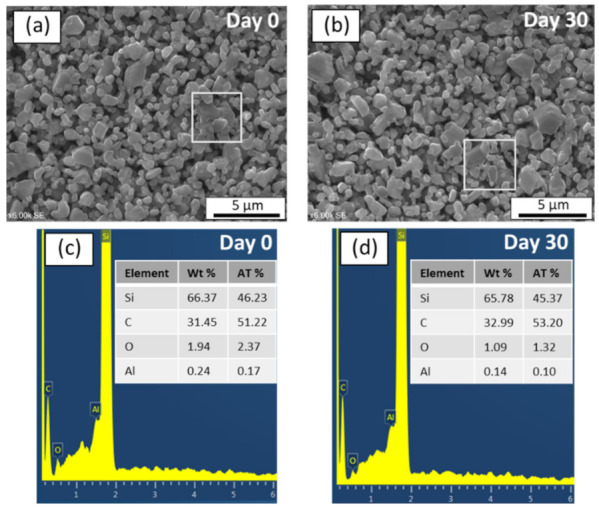
(**a**,**b**) SEM images of SiC membrane before and after acid corrosion, respectively and (**c**,**d**) Chemical composition of SiC membrane layer before and after acid corrosion, respectively.

In order to observe the surface morphology of the SiC membrane layers before and after acid corrosion tests, SEM images of the membrane surfaces are shown in Figure 9a,b. The SEM images show no clear difference or change on the membrane morphology after 30 days under corrosion test. Additionally, it is observed that there was no significant weight loss after 30 days. The EDX spectrums with chemical compositions of the membrane layers before and after acid resistance test were given in the Figure 9c,d, respectively. The elemental composition of the SiC membrane surface (highlighted in Figure 9a) before exposure to acid solution was: Si (66.37 wt%), C (31.45 wt%), O (1.94 wt%), and Al (0.24 wt%). After the 30-day test in acidic solution, the EDX spectrum of region highlighted in Figure 9b indicates that the membrane surface consisted of Si (65.78 wt%), C (32.99 wt%), O (1.09 wt%), and Al (0.14 wt%). As a result, there were no significant change observed in the composition of elements after immersion in acid solution for 30 days. These results give a clear indication that developed SiC membrane is also stable in an acidic environment. 

**Figure 10:**
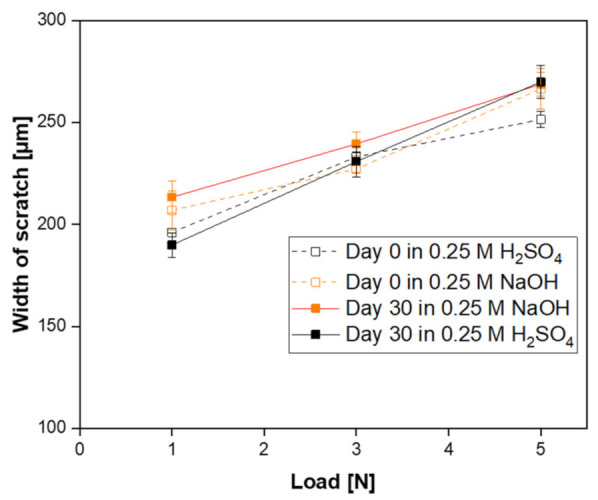
Width of scratch as a function of the applied loads for prepared SiC membranes before and after corrosion resistance test in alkali and acidic medium.

In addition, mechanical stability of the acid and alkali exposed SiC membrane samples were analyzed by means of scratch tests in order to detect possible changes in the membrane strength. The scratch widths of each membrane as a function of the applied load before and after acid and alkali corrosion test are shown in Figure 10. For all the samples, the scratch width increases with an increase of applied load. After 30 days of alkali exposure, no significant change in the scratch width of the membrane compared with an unexposed sample was observed for the three applied loads, indicating no variation in mechanical resistance. On the other hand, when the membrane was immersed in acid solution, the scratch width of the membrane remained unaffected with the load of 1N and 3N, but increased when 5N were applied, indicating a decrease in mechanical strength of the membrane. 

In the literature, there are several reports showing the corrosion resistance of SiC membranes [22,27,29,32,46,47]. For example, Jiang et al. [32] prepared SiC ceramic membrane using zeolite residue as sintering additive and exposed them to solutions of 20 vol% H_2_SO_4_ or 1 wt% NaOH at 90 °C over 300 h, respectively. When the membrane was immersed into NaOH solution, the bending strength of the membrane decreased from 45 MPa to 39 MPa in the first 46 h and remained stable over the next 300 h. Even though the mechanical strength decreased slightly, they have found that the pore size distribution of the membrane was unchanged. Moreover, the samples did not corrode after over 300 h of acid exposure. Wei et al. [27] reported that non-oxide SiC membranes was stable after 6 h of immersion of the membranes in 0.25 M NaOH solution and 0.25 M H_2_SO_4_ solution at 100 °C. In another study [47], glass-bonded SiC membranes subjected to pH solution of 3 or 11 up to 63 days at room temperature. After 63 days exposure, the flexural strength of the samples decreased from 80 MPa to 57 MPa in the pH 3 solution and to 48 MPa in the pH 11 solution. 

The SiC membranes developed in this study present good resistance under the exposure of alkali and acid solutions, also when compared to other studies from the literature. These results suggest that the new SiC membranes can be considered for water and wastewater treatments, particularly for those applications, which require frequent chemical cleaning of the fouled layer. One relevant example is the filtration of olive oil washing wastewater [48].

### 3.4. Filtration Performance of SiC Membrane 

**Figure 11:**
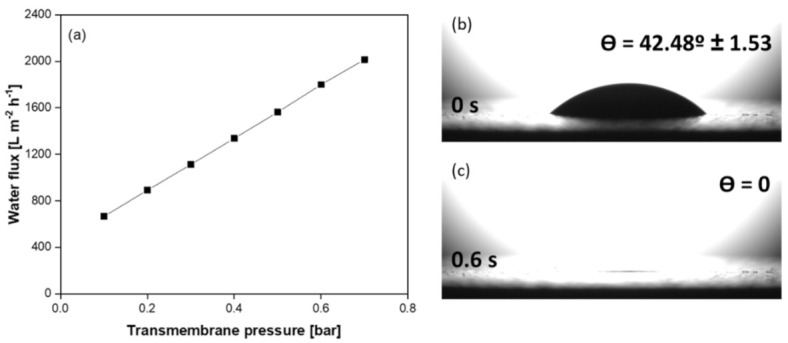
(**a**) Pure water flux as a function of applied transmembrane pressure for duplicated SiC membranes in a crossflow configuration and (**b**,**c**) The water/membrane initial contact angle image of SiC membrane and the contact angle image of SiC membrane when the water droplet reached to a steady position, respectively.

Pure water fluxes of the new SiC membrane deposited on multi-channeled SiC tubular support were analyzed. All experiments were repeated two times to assess reproducibility. Figure 11a shows the pure water flux of the membrane as a function of applied transmembrane pressure. As it can be seen from the graph, the flux and transmembrane pressure are linearly related as there is no fouling. The fluxes increased from 667 to 2014 L m^−2^ h^−1^ by raising the differential pressure from 0.1 to 0.7 bar. From the slope of the linear region the pure water permeability of the SiC membrane was calculated as 2252 L h^−1^ m^−2^ bar^−1^. The higher water permeance of the membrane can be due to the open porosity coupled to asymmetry of the membrane structure.

Membrane wettability and hydrophilic characteristics are crucial for highly efficient oil/water separation [49], as needed for the treatment of olive oil washing wastewater. Therefore, surface-wetting properties of the membrane were studied. For this aim, initial water contact angle of the as-prepared SiC membrane was analyzed. As can be seen in Figure 11b,c, the prepared SiC membrane exhibited good hydrophilicity with an initial water contact angle of 42.5°. This data indicates that the prepared membrane has high wetting characteristics for effective filtration. The water droplet could permeate into pores within 0.6 s. This very fast penetration time can be ascribed to permeable and highly porous structure of the prepared SiC membrane.

**Figure 12:**
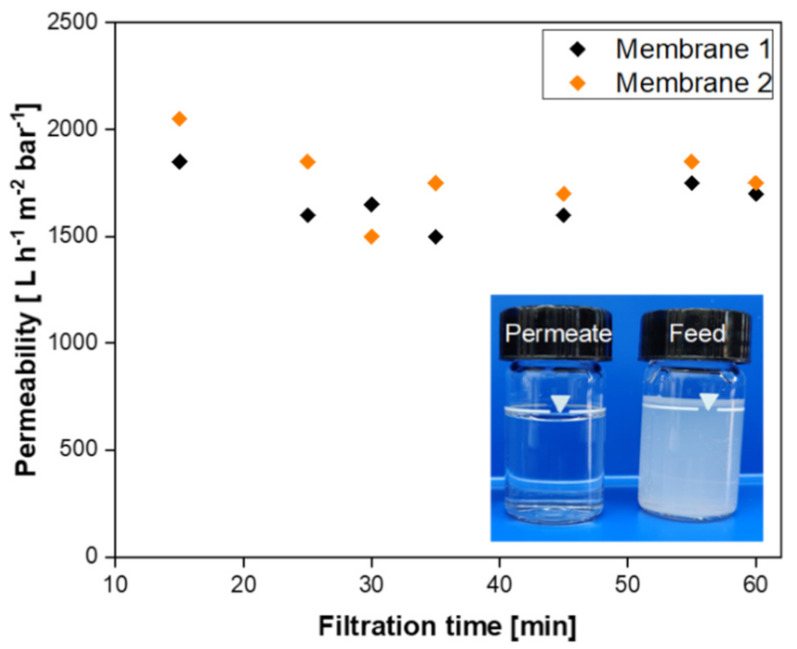
Duplicated permeability values as a function of filtration time for SiC membranes.

The performance of the as-prepared SiC membrane was evaluated by filtering a model olive oil washing wastewater. To this end, the olive oil/water emulsion was circulated into the system and the system was operated at constant flux of 150 L m^−2^ h^−1^ for 60 min. Figure 12 presents the permeability data obtained from these filtration experiments for two samples. As shown in the graph, the permeability of the duplicated membranes remained constant, meaning no fouling observed during filtration the olive oil-in-water emulsion. The characteristic of feed and permeate samples were analyzed in terms of oil and grease content. The oil and grease content in the feed (84 mg/L) was reduced to 0.18 mg/L in the permeate. According to these values, the oil and grease removal efficiency of the SiC membrane was calculated as 99.7%. As shown in the inset of Figure 12, the feed olive oil in water emulsion is milky white due to the presence of large amount of oil droplets. After separation by as-prepared SiC membrane, almost all droplets were removed and therefore the permeate is transparent. 

Table 1 reports the removal efficiencies of other ceramic membranes consisting of different materials and having difference geometry and pore size. The oil removal rates of SiC membranes and other ceramic membranes, with pore size of 0.2–0.7 µm, were in the range of 84–99%. Pan et al. [50] showed that high oil retention (99.1%) can be achieved by TiO_2_ membranes even with pore in the range 1.4–2.8 µm. This finding can be explained by the hydrophilicity of the TiO_2_ membrane, but also depends on the stability of the oil-in-water suspension. Even though these membranes showed similar oil removal rates, the permeability of the SiC membrane developed in this study was significantly higher than that of other SiC membranes [32,51,52,53], Al_2_O_3_ [54], ZrO_2_-Al_2_O_3_ [55], and TiO_2_ [50] membranes. The enhanced permeability is beneficial for reducing investment costs and footprint of the membrane installations, two crucial aspects for the application of ceramic membranes in the treatment of oily wastewater, as in the food industry or in the enhanced recovery of oil and gas. 

## 4. Conclusions

In this work, ceramic processing route for the development of a new silicon carbide membrane was studied by the aid of sintering additive and the prepared membranes were characterized in terms of their chemical and mechanical stability. Moreover, the performance of the final membrane was evaluated by filtering emulsified oil in water. The key findings are summarized as follows:

- The successful preparation of coating suspension containing two different α-SiC powders with the mixing ratio of 1:1 and 10^−4^ M aluminum nitrate nonahydrate as a sintering additive resulted in a homogeneous, defect-free, and smooth SiC membrane layer on macroporous multi-channeled SiC tubular support. With the use of aluminum nitrate nonahydrate, the sintering temperature was reduced approximately 200 °C with respect to the conventional SiC membrane fabrication. This decrease in the sintering temperature effectively reduces the cost of production of SiC membrane. The developed membrane has a narrow pore size distribution with an average pore size of 0.35 µm, which indicates the membranes are suitable for microfiltration.
- The prepared SiC membrane demonstrated a good chemical resistance in both alkali and acid environments for 30 days. In both cases, pore size distributions, surface morphologies, and elemental compositions of the membranes were not changed significantly before and after exposure to a 30-day corrosion resistance test. Moreover, no change in the membrane strength was detected after corrosion tests, which shows that the developed membrane has good mechanical resistance.
- The resultant SiC membrane showed outstanding water permeability (2252 L h^−1^ m^−2^ bar^−1^) and remarkable separation performance (99.7%) for oil/water emulsion with excellent permeability. Therefore, this membrane has the potential for application in the treatment of oily wastewater streams.

## Figures and Tables

**Table 1:** Comparison of the oil removal performance of different ceramic membranes reported in the literature.

Membrane Material	Membrane Geometry	Pore Size [µm]	Operating Parameters	Oil Rejection (%)	Ref.
SiC	Disc	0.4	Constant permeate flux: 162 LMH	98.52	[32]
			Cross-flow velocity: 1.3 m/s		
			TMP: 0.5 bar		
SiC	Multi-channeled tubular	0.269–0.282	Cross-flow velocity: 0.5 m/s TMP: 1.5 bar	93.8	[51]
SiC	Multi-channeled tubular	0.269–0.282	Constant permeate flux: 67 LMH Cross-flow velocity: 2 m/s	84	[52]
SiC	Hollow fiber	0.71	Constant permeate flux: 103.9 LMH	93.5	[53]
			Cross-flow velocity: 0.15 m/s TMP: 0.25 bar		
Al_2_O_3_	Multi-channeled tubular	0.2	Cross-flow velocity: 2.25 m/s TMP: 1.25 bar	85	[54]
ZrO_2_-Al_2_O_3_	Tubular	0.2	Cross-flow velocity: 5 m/s TMP: 0.16 bar	97.8	[55]
TiO_2_	Tubular	1.4–2.8	Constant permeate flux: 599.7 LMH Cross-flow velocity: 1.13 m/s	99.1	[46]
This work	Multi-channeled tubular	0.35	Constant permeate flux: 150 LMH Cross-flow: 1527 L/h TMP: 0.08 bar	99.7	

TMP: transmembrane pressure, LMH: flux unit (L/h/m^2^).
